# A Profile and Three-Year Follow-Up of Patients with Basal Cell Carcinoma in the Western Cape, South Africa

**DOI:** 10.1155/2022/8443867

**Published:** 2022-05-05

**Authors:** J. C. Gallo, J. W. Schneider, J. de Wet, K. Moxley, H. F. Jordaan, W. I. Visser, B. Tod

**Affiliations:** ^1^Division of Dermatology, Department of Medicine, Tygerberg Academic Hospital, Stellenbosch University, Cape Town, South Africa; ^2^Division of Anatomical Pathology, Department of Pathology, National Health Laboratory Service, Tygerberg Academic Hospital, Stellenbosch University, Cape Town, South Africa; ^3^Registrar Research Support Office, Research Development and Support Division, Faculty of Medicine and Health Sciences, Stellenbosch University, Cape Town, South Africa

## Abstract

**Background:**

Basal cell carcinoma (BCC) is an important malignancy in sub-Saharan Africa. There is a paucity of data regarding BCC in South Africa.

**Aims:**

To describe the clinicopathological features of patients presenting with BCC in a cohort of South African patients.

**Methods:**

This retrospective descriptive study reviewed the medical records of 149 patients with BCC who attended the dermatology clinic at Tygerberg Academic Hospital from September 2015 to August 2016. Demographic and clinical data of those patients with histologically proven BCC were retrieved from clinical records. The data included the assessment for BCC recurrence after three years (September 2016–August 2019).

**Results:**

Of 390 patients, 155 (39.7%) had histologically confirmed BCCs. Complete medical records were available for 149 of these patients, and most were male (55.7%) and white (85.9%) with a median age of 70 years. Most patients had their BCC lesions for 12 months (43.1%) before diagnosis. BCCs were mostly located on the head and neck area (58.1%). In most patients (72.0%), a diagnostic punch biopsy confirmed BCC. Plastic surgeons subsequently excised the BCC lesions in 74.0% of these patients. The most common histological subtype was nodular BCC (74.0%). The National Comprehensive Cancer Network (NCCN) risk of recurrence was approximately evenly distributed between high- (54.1%) and low-risk groups (45.9%). The major high-risk feature was the location (36.6%). Histologically confirmed BCC recurrence occurred in 9 of the 149 patients (3.7%) over three years.

**Conclusions:**

BCC represents a high burden of disease in our setting. Compared to existing studies, the BCCs in this study are clinically and histologically similar to international reports.

## 1. Introduction

Basal cell carcinoma (BCC) is a type of keratinocyte cancer (KC) that arises from basal epidermal keratinocytes. BCC is the commonest malignancy of the skin and the commonest human malignancy worldwide [1–3]. BCC represents approximately 70–80% of all skin carcinomas, making it the most prevalent cancer in many countries [2, 4, 5].

BCC is rarely fatal (mortality rate <0.1%), but localized tissue invasion may induce considerable morbidity. Most BCC lesions occur on cosmetically sensitive areas such as the scalp, neck, and face. Destructive invasion by BCC or therapeutic interventions may cause functional and cosmetic problems [4, 5].

BCC management involves consultations by primary healthcare physicians or dermatologists, diagnostic biopsies, histopathology review, formal surgical excision in the dermatology office, referral for Mohs micrographic surgery (MMS), or referral for plastic and reconstructive surgery and, in selected cases, radiation therapy. Patients require regular follow-up visits to monitor for BCC recurrence or the development of new skin cancers. Delayed diagnosis is a significant challenge in low- and middle-income countries. BCC adds a high-cost burden and strain on the health care system [4, 6].

BCC has been extensively studied in Australia, the United States, and Europe. These studies confirmed wide variation in BCC incidence, with the highest rates in Australia and the lowest rates in parts of Africa [4, 7]. Higher incidence is related to decreasing latitude, intermittent solar ultraviolet radiation (UVR) exposure, and more accurate reporting practices [4]. Little is known regarding BCC in populations in low- and middle-income countries such as South Africa (SA).

SA has one of the world's largest human immunodeficiency virus (HIV) epidemics [8]. HIV-infected patients have a 3- to 5-fold increased risk of developing KCs [7, 9, 10]. SA has high levels of solar UVR due to its geographical location [9]. Furthermore, skin cancer rates in SA have been increasing [9, 10]. Incomplete reporting to the National Cancer Registry explains the limited data about BCC in SA, making it difficult to monitor the national statistics compared to other countries [10]. The paucity of clinicopathological data for BCC in SA justifies research to address this knowledge gap and to create greater awareness and a better understanding of BCC in SA. Such studies can facilitate educational and prevention campaigns to assist in curbing the burden of BCC.

## 2. Materials and Methods

### 2.1. Study Design

This retrospective descriptive study included patients with histologically proven BCC who attended the dermatology clinic at Tygerberg Academic Hospital (TAH) between 1 September 2015 and 31 August 2016. The study included a three-year follow-up of patients (until 31 August 2019) to assess for BCC recurrence.

### 2.2. Study Setting

TAH is a publicly funded academic tertiary level hospital in Cape Town (Western Cape province, SA) with a large drainage area from primary and secondary health care facilities. TAH serves a population of low- to middle-income status. The dermatology clinic is operated by consultant dermatologists, registrar dermatologists, and nurses. The unit is medically focused, where excisions and cautery and electrodesiccation (C&E) of low-risk tumours are performed. High-risk and surgically challenging tumours are referred to the Division of Plastic and Reconstructive Surgery at TAH for excision.

### 2.3. Study Sample

During the study period, 696 biopsies from 390 patients were performed on clinically suspected skin cancer [11]. We included patients older than 18 years with histologically confirmed BCC. Patients with incomplete medical records and those lost to follow-up were excluded. The final sample size for data collection was therefore 149.

### 2.4. Data Collection

Demographic data were obtained as secondary data from a previous study (HREC/REF: U16/10/028) [11]. Data about relevant medical history, previous treatments for BCC, tumour diameter and location, the procedure performed, and any BCC recurrence were retrieved for each case from records on the TAH Enterprise Content Management (ECM) system. Histopathology results were obtained from the National Health Laboratory Service database. BCC recurrence was identified by reviewing patient records on the ECM from 1 September 2016 to 31 August 2019. Recurrence was defined as “possible local recurrence” when histologically confirmed BCC arose within the scar of a previously excised BCC [12]. The principal investigator (JG) collated the anonymised data in a Microsoft Excel spreadsheet.

### 2.5. Patient Characteristics

Patient records did not include Fitzpatrick phototypes. Therefore, we used the population group as a crude indication of phototype^*∗*^. A hot-deck imputation method was used when the population group was not indicated [11, 13]. Patients were recorded as immunosuppressed if they had HIV, had a current malignancy (excluding KC), had a solid organ transplant, or used immunosuppressive medication.

### 2.6. Diagnosis of BCC

Dermatology trainees and consultants from the Division of Dermatology at TAH performed biopsies and other surgical procedures. In some cases, patients had more than one BCC, requiring multiple biopsies at the same visit.

The National Comprehensive Cancer Network (NCCN) BCC guidelines define criteria for high recurrence risk to include specific tumour locations and sizes, ill-defined clinical borders, recurrences, tumours arising in radiotherapy fields, aggressive histological growth patterns, perineural involvement, and patient immunosuppression [14].

A pathologist (JWS) and JG retrieved and reviewed histopathology sections of cases where the histopathology reports did not include adequate information regarding diagnosis, histological growth pattern, or perineural involvement. JWS reviewed all BCC specimens and categorized them according to the 4th edition World Health Organisation (WHO) classification guideline for tumours [15].

### 2.7. Data Analysis

Continuous variables were summarised as median with interquartile range (IQR) for non-normal data. Categorical variables were summarised as frequencies and percentages.

### 2.8. Ethical Considerations

The study was approved by the Stellenbosch University Health Research Ethics Committee (SUHREC) (HREC/REF: S19/04/070). All data were anonymised to ensure the confidentiality of participants' personal information.

## 3. Results

Of 390 patients biopsied for clinically suspected KC, a total of 155 had a confirmed diagnosis of BCC (39.7%). Six patients with incomplete medical records were excluded. Fifty-six patients (36.1%) presented with multiple BCCs. Demographic and clinical characteristics of 149 patients meeting inclusion criteria are summarised in Table 1. The median age (interquartile range, IQR) was 70 (61–78) years.

In the mixed ancestry group^*∗*^ (*n* = 18), 13 patients (72.2%) had high-risk features. In contrast, the white population group^*∗*^ (*n* = 128) had high-risk features in 67 patients (52.3%). There were a 2.8 (95% CI: 0.97 to 7.86) odds of having a higher risk profile in the mixed ancestry group^*∗*^. However, this finding was not statistically significant. The single black African^*∗*^ patient was at high-risk due to immunosuppression from HIV infection and had oculocutaneous albinism (OCA) and, therefore, presumably lightly pigmented skin. The Indian/Asian^*∗*^ patient had a high-risk feature due to the BCC occurring in a high-risk location.

Risk factors for BCC were poorly documented. In 48 cases with this information, the commonest risk factor was chronic sun exposure (*n* = 8), of which 6 cases were occupational. Sun exposure was only captured if it was reported in the patient's clinical notes. The histological assessment confirmed the presence of solar elastosis in 161 cases (65.4%), an absence thereof in 9 cases (3.7%), and “unable to assess” in 76 cases (31.0%). Where solar elastosis could be histologically assessed, it was found in 94.7% of cases. Thus, it appears that chronic sun exposure was significantly underreported.

Immunosuppression was reported in only 7 patients who collectively had 17 biopsies performed. Two patients were HIV-positive, of whom one had previous PUVA treatment, and the other had OCA. One patient took methotrexate therapy for psoriasis. Three patients had cancer elsewhere and one was a renal transplant recipient on immunosuppressive medication.

A total of 246 BCCs were diagnosed amongst 149 patients and the tumour characteristics are summarised in Table 2. Combined high-risk histological subtypes, including micronodular, morpheaform, and basosquamous subtypes, amounted to 24 cases (9.8%). From the pigmented BCC group (*n* = 22), 8 cases were considered as high-risk BCCs, with the most common high-risk feature being increased lesion size (*n* = 6). Closer examination of the pigmented BCC group (*n* = 22) in the current study revealed that 15 arose in white patients^*∗*^ and the remainder (*n* = 7) from other phototypes (Indian/Asian and mixed ancestry^*∗*^). Of biopsies from presumably more pigmented phototypes (all skin types excluding white patients^*∗*^), a third were pigmented BCCs. Ulceration was noted in 54 cases (22.0%). None of our cases showed perineural invasion.

**Figure 1:**
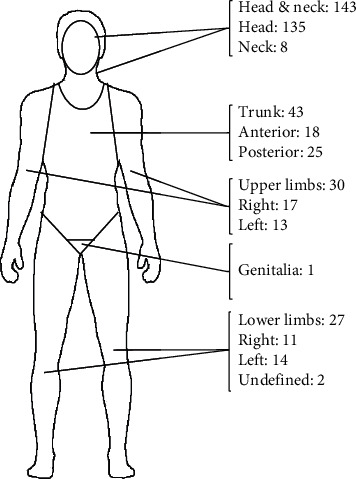
Distribution of basal cell carcinoma tumours on the body (*n* = 244, *n* = 2 location not stated).

**Figure 2:**
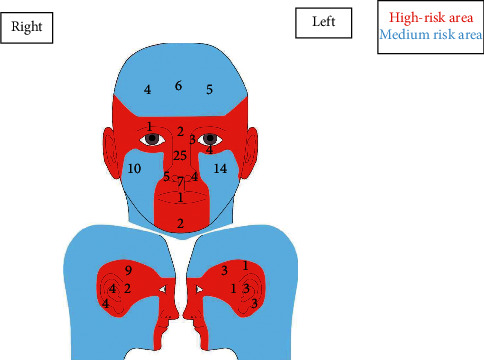
Distribution of basal cell carcinoma tumours on the face (*n* = 126). The area in red is the high-risk mask area of the face (*n* = 87), and the blue area signifies the rest of the face, which is a medium risk (*n* = 39).

Figures 1 and 2 depict BCC location in the cohort. BCCs were more common on the left side of the face (*n* = 47) compared to the right side of the face (*n* = 39) (Figure 2). The side of the face was not specified in 31 cases, and the remaining 9 cases were in the midline. The nose was the most common area of the face involved (*n* = 43), followed by the cheeks (*n* = 24).

Fifty-six (22.8%) patients were treated by double-cycle C&E or formal excision at the dermatology clinic. NCCN risk stratification, final treatment modalities, and recurrences are presented in Table 3. One patient was referred to an ophthalmologist for excision of a lesion close to the eyelid margin (*n* = 1). Two patients, one with a high-risk basosquamous lesion and the other with a squamous cell carcinoma elsewhere, were referred to a multidisciplinary skin cancer clinic. Excision by a plastic and reconstructive surgeon resulted in 6 recurrences (of 182 cases treated, 3.3%). One patient in the “other treatments” group had nodal metastases. This lesion was a high-risk basosquamous carcinoma referred to the multidisciplinary skin cancer clinic for surgical nodal clearance.

Recurrence was clinically suspected in 24 cases, although it was histologically proven in only 9 cases (3.7%) over the three-year follow-up period. Most recurrences occurred 12–24 months after the initial event (*n* = 5). There were 8 cases of local recurrence and 1 case of metastatic spread to draining lymph nodes (the basosquamous carcinoma mentioned above). New BCC lesions in locations other than the index BCC lesion occurred in 83 cases (33.7%).

## 4. Discussion

BCC is a common malignancy with significant morbidity and health economic implications. A large body of research on BCC is available. However, little local data exist about BCC in the South African context. The purpose of this retrospective descriptive study was to determine the prevalence, clinicopathological features, and recurrence of BCC in a cohort of patients presenting with histologically proven BCC at a single institution.

### 4.1. Demographic Characteristics

The overall prevalence of BCC in patients with clinically suspected skin cancer in this study sample was 39.7%. In keeping with international trends, most patients with BCC were male (55.7%) and white^*∗*^ (85.9%) with a median age of 70 years [2, 16, 17]. Data regarding BCC in other population groups in SA are sparse [18, 19]. In the United States, studies show that skin cancer occurs less frequently in darker pigmented skin types [19, 20]. Our study is in keeping with this trend.

Skin cancer outcomes tend to be worse in patients with pigmented skin types [21]. Our findings echo this observation given the higher risk profile of the mixed ancestry group^*∗*^ compared to the white population group^*∗*^ (72.2% versus 52.3% with high-risk features). The prevalence of this outcome should be reexamined in research statistically powered for assessing this outcome, as it has important clinical implications. This study could not provide causes for the possible risk-related differences between these two groups, and further studies could investigate this question.

### 4.2. Risk Factors for the Development of BCC

Although SA also has a high rate of year-round solar UVR exposure, the population composition is very different from other countries in the southern hemisphere with high BCC rates, such as Australia [19]. Solar elastosis was observed in most cases, suggesting significant sun damage in these patients. Regarding immunosuppression, SA has one of the highest rates of HIV in the world at an estimated 13% prevalence, and approximately, 7-8 million people were living with HIV in 2020 [8]. Both HIV-positive patients in our study had other risk factors for BCC (OCA and previous PUVA exposure). SA theoretically has a population uniquely predisposed to BCC due to high levels of UVR coupled with a large portion of the population being immunosuppressed due to HIV. Further studies are required to fully understand this.

### 4.3. Duration of BCC Prior to Diagnosis

Delayed BCC diagnosis has been linked to recurrence and metastatic risk [22]. Most of the patients in this study presented to the dermatology clinic having had BCC for more than 12 months. Simultaneously, most cases involved conspicuous areas such as the face, and there was a high proportion of large, locally advanced lesions at presentation (18.7% were larger than 10 mm). This suggests a lack of awareness about the clinical signs of BCC. There is a need to educate patients and health-care workers to identify BCC and seek professional medical care promptly, thereby improving surgical outcomes and limiting disfigurement due to larger tumours [2, 22].

### 4.4. Histology of BCC

There are several described histological subtypes of BCC [15, 23]. The World Health Organisation (WHO) classifies the various BCC subtypes according to their different growth patterns [15]. Nodular, superficial, and pigmented BCC subtypes are generally considered less aggressive, whereas micronodular, morpheaform, basosquamous, and infiltrating subtypes are more aggressive. Recent research has highlighted a different trend for the involvement of lateral or deep margins according to different histological subtypes [23]. Lateral involvement is more frequent for the morpheaform subtype whereas the deep margin is more involved in the nodular subtype [23]. In this study, the most common BCC subtype was nodular (74.0%), in line with international literature [2, 23]. The more aggressive histological subtypes (*n* = 24, 9.8%) are deemed high-risk for recurrence using the NCCN guidelines [14].

Pigmented BCCs are considered a less aggressive histological subtype [24]. There was a high proportion of other high-risk features among pigmented BCCs in this cohort. The most common high-risk feature was increased clinical lesion size at the time of diagnosis, highlighting the diagnostic delay in these challenging cases. In previous studies, pigmented BCCs have been shown to predominate in darker pigmented skin types [22, 24]. Pigmented BCCs were more prevalent in pigmented skin types in this cohort. This is clinically relevant as pigmented BCCs may be misdiagnosed as seborrheic keratoses or melanocytic naevi, for example, in darker skin types. Dermoscopy can assist in differentiating such lesions from BCC [25]. Clinicians should be aware of this important differential diagnosis in patients with darker skin types.

### 4.5. National Comprehensive Cancer Network (NCCN) Risk Stratification

Our cohort was stratified utilizing the NCCN risk for recurrence stratification, defining a large proportion of patients as high-risk for recurrence (*n* = 133, 54.0%), mostly due to BCC location. A low recurrence rate was observed; however, limitations in the methodology mean that this has probably been underreported.

### 4.6. BCC Treatment

The goal of BCC treatment is a cure with maximum preservation of function, cosmesis, and low complication rates [3, 26, 27]. The NCCN guidelines for the surgical treatment of BCC recommend that low-risk BCCs less than 2 cm in diameter be excised with a 4 mm clinical margin. MMS is the gold standard treatment for high-risk BCCs as it offers the highest cure rate [27–29]. A study evaluating MMS for BCC treatment in SA echoed international data and showed that MMS offers the highest cure rates with a low recurrence rate (0.1%) [29]. MMS is not a treatment option at TAH.

Double-cycle C&E produced good results for our cohort, with seemingly low recurrence rates. C&E is indicated for the treatment of small, low-risk nonfacial BCCs and patients who are not surgical candidates [26]. Although not recommended by international guidelines, C&E can be considered in low-resource settings for the treatment of high-risk BCCs, since it is a convenient and cost-effective procedure. This occurs in carefully selected patients at TAH. BCC recurrence in high-risk areas treated with C&E is reported to be significantly higher compared to surgical alternatives (6.9% versus 3.8% respectively) [30, 31]. High-risk BCCs treated with C&E require more rigorous follow-up for recurrence, which could result in an increased demand on resources and loss to follow-up.

Training primary and secondary healthcare workers to diagnose and treat low-risk BCCs with C&E could offer better treatment outcomes than long delays in referral to a tertiary dermatology centre. Surgical training of dermatology trainees to excise high-risk BCCs would necessitate fewer referrals to plastic and reconstructive surgery. Further studies are required to assess the need for an adapted BCC risk stratification in resource-limited environments and more accurately evaluate the success of double-cycle C&E in selected high-risk lesions.

### 4.7. BCC Recurrence

Possible recurrence was observed in 9 patients (3.7%). Six patients with recurrent BCC were initially treated for their primary BCC by surgical excision, and the majority occurred 12–24 months after the initial event. These results are in keeping with international trends of BCC recurrence rates [3, 22]. However, patients were only followed up over three years, and cases could have recurred after this time or been lost to follow-up. A randomised control trial with a 10-year follow-up period found that over 50% of BCC recurrences occur after five years [30]. Therefore, the three-year follow-up period in this study could lead to a misleadingly low recurrence rate.

### 4.8. Study Limitations

A limitation of this retrospective study is that our data depended on the accuracy of the clinical records. The small size of the study was also a limitation and further research including a larger cohort of patients with BCC is required to profile this group of patients in our setting more accurately. NCCN risk factors for recurrence such as tumour size and clinical borders could not be comprehensively studied due to incomplete data in the majority of clinical records. The use of population group as a proxy for Fitzpatrick phototype means that our findings in this respect must be viewed with circumspection^*∗*^. In most cases, only tumour tissue obtained by curettage or punch biopsy was available to assess histological BCC subtypes and perineural invasion. Limited amounts of tumour tissue may not be representative of the entire BCC, potentially compromising conclusions about BCC subtypes and recurrence. The recurrence assessment was limited by the retrospective study design, short follow-up period, incomplete clinical records, and patients being lost to follow-up. Missed recurrences might distort the recurrence rate after double-cycle C&E. Prospective research with a longer follow-up period might assess the recurrence rate more accurately.

## 5. Conclusion

This study showed that BCC in the South African context is clinically and histologically similar to BCC described in other geographical areas. BCC is the commonest cancer observed in our dermatology clinic, corresponding with a high disease burden. Surgical training of primary and secondary level doctors to excise low-risk BCCs could reduce the disease burden and referral load. Adaptation of referral protocols to plastic and reconstructive surgery in low-resource settings may be prudent. This study emphasises the need for prevention and early diagnostic strategies that might mitigate the rising incidence of BCC in the local context. Furthermore, early surgical management of BCCs will improve patients' outcomes and reduce the risk of future complications.

## Figures and Tables

**Table 1:** Demographic and clinical characteristics of patients with BCC (*N* = 149).

Variable	Frequency, *n*	Percentage, %
*Gender*		
Male	83	55.7
Female	66	44.3

*Population group*		
White^*∗*^	128	85.9
Mixed ancestry^*∗*^	18	12.1
Black African^*∗*^	1	0.7
Indian/Asian^*∗*^	1	0.7
Not known	1	0.7

*Risk factors*		
Chronic sun exposure	8	5.4
Immunosuppression	5	3.4
Site of prior radiation	3	2.0
Oculocutaneous albinism (OCA)	1	0.7
Immunosuppression and OCA	1	0.7
Immunosuppression and previous psoralen ultraviolet a (PUVA) therapy	1	0.7
None	29	19.5
Not known	101	67.8

*Previous history of skin cancer*		
Unspecified KC	43	28.9
Basal cell carcinoma	24	16.1
Squamous cell carcinoma	4	2.7
Cutaneous melanoma	3	2.0
KC and cutaneous melanoma	8	5.4
KC and other skin cancers	2	1.3
None	11	7.4
Not known	54	36.2

**Table 2:** Clinical and pathological data of all incident basal cell carcinomas (*N* = 246 biopsy samples).

Variable	Frequency, *n*	Percentage, %
*Clinical diameter (mm)*		
<10	28	11.4
10–19	27	11
≥20	19	7.7
Not stated in clinical notes	172	69.9

*Duration (months)*		
<6 months	67	27.2
6–12 months	51	20.7
>12 months	106	43.1
Participant unsure	9	5.3
Not stated in clinical notes	13	3.7

*Diagnostic procedure*		
Punch biopsy	177	72.0
Double-cycle C&E	45	18.3
Excisional biopsy	21	8.5
Incisional biopsy	2	0.8
Shave biopsy	2	0.8

*BCC subtype*		
Nodular	182	74.0
Pigmented	22	8.9
Micronodular	21	8.5
Superficial	5	2.0
Morpheaform	2	0.8
Basosquamous	1	0.4
Unable to assess	13	5.3

*NCCN risk stratification*		
High-risk	133	54.1
Low-risk	113	45.9

*Number of high-risk features*		
1 high-risk feature	107	43.5
2 high-risk features	22	8.9
3 high-risk features	4	1.6

*High-risk features*		
Location	90	36.6
Size	24	9.8
Aggressive growth pattern	24	9.8
Immunosuppression	17	6.9
Site of prior radiation	5	2.0
Recurrent BCCs	3	1.2

**Table 3:** NCCN risk stratification and final treatment modalities for BCC lesions (*N* = 246).

Treatment modality, *n* (%)	Total high- and low-risk (*N* = 246)	Risk stratification	
		High-risk (*n* = 135)	Low-risk (*n* = 111)
Excision by plastic and reconstructive surgery	182 (74.0)	109 (44.3) (3^*∗∗*^)	73 (29.7) (3^*∗∗*^)
Double-cycle C&E at dermatology	37 (15.0)	18 (7.3)	19 (7.7) (2^*∗∗*^)
Excision at dermatology	19 (7.7)	4 (1.6)	15 (6.1)
Other treatments	3 (1.2)	2 (0.8) (1^*∗∗*^)	1 (0.4)
Loss to follow-up	5 (2.0)	2 (0.8)	3 (1.2)

(*n*^*∗∗*^) represents the number of recurrent cases in a specific treatment modality.

## Data Availability

Data can be available on request. The requesting party can contact the corresponding author.

## Additional Points

Variations in the epidemiology and clinical features of BCC significantly correlate with patients' Fitzpatrick phototypes, justifying the inclusion of this data [21]. While the population group (a social construct) is not reliably correlated with the phototype, it is used in this study as a crude proxy.
